# Lean body mass and stroke volume, a sex issue

**DOI:** 10.3389/fneur.2024.1443356

**Published:** 2025-01-22

**Authors:** Bertil Delsaut, Anissa Abderrakib, Noémie Ligot, Gilles Naeije

**Affiliations:** ^1^Department of Neurology, Tivoli Hospital, La Louvière, Belgium; ^2^Department of Neurology, CUB Hôpital Erasme, Université libre de Bruxelles (ULB), Brussels, Belgium

**Keywords:** ischemic stroke, lean body mass, sex, core, penumbra

## Abstract

**Introduction:**

Large vessel occlusions (LVO) account for over 60% of stroke-related mortality and disability. Lean body mass (LBM) represents metabolically active body tissue and has been associated with reduced mortality. This study aimed to investigate whether body composition influences LVO stroke perfusion volumes and whether this effect is sex-specific.

**Methods:**

Data were retrospectively collected from all patients admitted between January 2017 and January 2022 with LVO ischemic stroke at the Erasmus Hospital (Brussels), for whom anthropometric and perfusion data were available. Body mass index (BMI) and LBM were calculated using, respectively, the Quetelet’s and the James’ formula. Correlations between body composition and stroke volumes were investigated using Spearman correlations.

**Results:**

A total of 152 patients were included in this study. Mean age 72 ± 14y, female ratio 62.5%, core volume 26 ± 38 mL, penumbra volume 104 ± 61 mL. LBM correlated significantly with stroke volumes (penumbra and core) in the entire group (core: *p* = 0.001; penumbra: *p* = 0.001). There was a significant sex-effect, with a significant correlation observed only in women (core: *p* = 0.008; penumbra: *p* = 0.007). BMI did not correlate with perfusion volumes at the group level nor at the sex-level.

**Conclusion:**

LBM significantly impacts LVO stroke volumes, but this effect is observed only in women. LBM may serve as a superior indicator of body composition compared to BMI.

## Introduction

Large vessel occlusions of the anterior circulation (LVO) account for over 60% of stroke-related mortality and disability (1, 2). In stroke, sexes are not equals. Women bear a disproportionate burden from stroke compared to men: women display higher rates of large territorial infarcts, higher mortality, are less likely to achieve functional independence after ischemic stroke in general and after endovascular treatment (EVT) for LVO in particular. Those differences are not entirely accounted for the average older age at stroke onset, the elevated pre-stroke disability level or the higher prevalence of cardio-embolic stroke that are associated with the female sex (3–5) hinting that sex-specific factors may influence LVO outcomes.

Patients with LVO stand to derive optimal benefit from thrombectomy when presenting with a small core infarction and a substantial volume of salvageable brain tissue at the time of baseline imaging evaluation (6). These conditions exhibit a positive correlation with enhanced collateral circulation at both the arterial and cerebral brain tissue levels (7), thereby underscoring their significant role in predicting favorable outcomes in thrombectomy intervention. In women, a discernible paradox is observed in terms of prognosis following an LVO. Despite exhibiting ostensibly superior collaterals (8), diminished ischemic stroke core volumes and better mismatch ratios (MMR) between penumbra and core volumes compared to their male counterparts (9), the prognostic outcomes for women following an LVO are notably poorer. One underexplored explanation may lie in the difference in body composition between male and female sexes. The body mass, indexed by the body mass index (BMI), is composed of the lean body mass (LBM), that comprises metabolically active tissues, including water, skeletal and smooth muscle mass and bone (10) and the fat mass (FM). Women have a lower proportion of LBM than men. Epidemiologically, an excess of FM is associated with greater cardiovascular morbidity (11) while higher LBM confers lower cardiovascular risk in both sexes (12) and is a protective factor for ischemic stroke (13). However, in female individuals, it has been observed that a heightened level of FM exerts a distinct mitigating effect on cardiovascular disease risk, irrespective of their level of LBM (12). These sex differences may contribute to the obesity paradox found in some studies that described better stroke and cardiovascular outcomes in patients with high BMI (14–17) while others fail to find such association (18). Similarly, in LVO, higher salvageable penumbra brain tissue was associated with a higher BMI in the data from The Acute STroke Registry and Analysis of Lausanne (19). In those studies, BMI was analysed as a whole and no distinctions were made between LBM, FM nor sexes which have blurred potentially relevant associations.

Here, we aimed to clarify the effects of gender and LBM on LVO strokes volumes and outcome in a large cohort of LVO using validated anthropometric prediction equations and perfusion volume assessment.

## Subjects and method

### Population

The studied population is derived from the stroke registry of Erasmus Hospital in Brussels (Belgium) where all case of acute stroke since January 2015 are recorded, and our analysis included patients admitted between January 2017 and April 2022. The inclusion criteria were as follows: confirmed LVO-stroke, performance of a perfusion-CT during the acute stroke phase, recorded weight and height, and age > 18 years. Data were collected retrospectively from the patients’ medical records.

BMI was calculated using Quetelet’s formula. LBM was determined using James’ formula (20):


$$BMI=\frac{\mathrm{weight}\;\left(\mathrm{kg}\right)}{{\left(\mathrm{height}\;\left(m\right)\right)}^{2}}$$

$$LBM\;\left(\mathrm{men}\right)=1,1\times \mathrm{weight}\left(\mathrm{kg}\right)-128\times {\left(\frac{\mathrm{weight}\;\left(\mathrm{kg}\right)}{\mathrm{height}\;\left(\mathrm{cm}\right)}\right)}^{2}$$

$$LBM\;\left(\mathrm{women}\right)=1,07\times \mathrm{weight}\left(\mathrm{kg}\right)-148\times {\left(\frac{\mathrm{weight}\;\left(\mathrm{kg}\right)}{\mathrm{height}\;\left(\mathrm{cm}\right)}\right)}^{2}$$

### Imaging

Pre-interventional imaging included non-contrast CT, CT angiography and CT perfusion (CTP). Ischemic core was defined as brain volume with cerebral blood flow (CBF) under 30% of the CBF of the homologous zone in the contralateral hemisphere. Ischemic penumbra was defined as brain volumes where the T_max_ of contrast product arrival exceeded 6 s. Those volume were automatically computed with the Rapid software (21) on which the cut-offs were based on (22).

### Statistical analysis

Clinical characteristics were reported using descriptive statistics. Correlations between body composition and stroke volumes were investigated using Spearman correlations. A Linear regression was performed for parameters that correlated with LBM. Finally, to further characterize the relationship between sex, stroke volumes, outcome and LBM, a median-split analysis of the LBM was performed as in (23). For group comparisons, when variables were continuous, a Mann–Whitney U test was applied. When they were discrete, a *χ*^2^ test was applied.

### Ethics

The study was reviewed and approved by the Ethics Committee of Erasmus Hospital, Route de Lennik, 808, Brussels, Belgium.

Due to the retrospective and non-interventional nature of this study, written informed consent for participation was not required for this study in accordance with the national legislation and the institutional requirement of the Ethics Committee of Erasmus Hospital, Route de Lennik, 808, Brussels, Belgium.

All methods were performed in accordance with the relevant guidelines and regulations.

## Results

### Population

**Figure 1:**
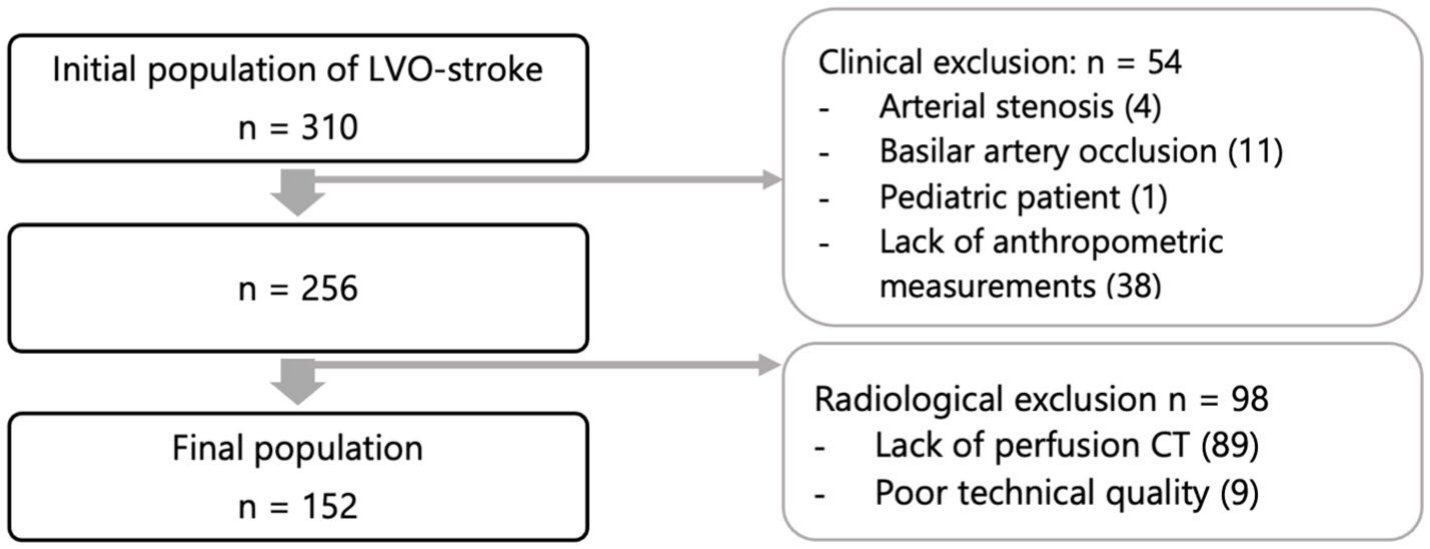
Flow chart.

Of an initial population of 310 LVO, 152 patients were included in the final analysis (Figure 1). Mean age of patients was 72 years old (62.5% female). Population characteristics are detailed in Table 1.

**Table 1:** Data summary.

			Entire group	Women	Men
Age (y)	mean ± sd		72 ± 14	72 ± 14	70 ± 14
BMI (kg/m^2^)	mean ± sd		26.3 ± 4.1	26 ± 4	26.8 ± 3.7
LBM (kg)	mean ± sd median		52.2 ± 8.5 51.2	47.5 ± 5.3 47.4	60 ± 7.2 59
Admission NIHSS (*n* = 151)	mean ± sd		15 ± 8	15 ± 8	15 ± 7
Discharge NIHSS (*n* = 135)	mean ± sd		7 ± 7	7 ± 8	7 ± 7
Core (ml)	mean ± sd		26 ± 38	21.9 ± 31.6	33.8 ± 45.2
Penumbra (ml)	mean ± sd		104 ± 61	96.39 ± 54.8	117 ± 69.1
Mismatch ratio			178.5	217.7	113.2
Known atrial fibrillation	*n* (%)		32 (21.1)	17 (17.9)	15 (26.3)
HTA	*n* (%)		110 (72.4)	68 (71.6)	42 (73.7)
Dyslipidaemia	*n* (%)		100 (65.8)	58 (61.1)	42 (73.7)
Type 2 Diabetes	*n* (%)		33 (21.7)	16 (16.8)	17 (29.8)
Smoke	*n* (%)		56 (36.8)	28 (29.5)	28 (49.1)
mRS at 3 months (*n* = 119)	*n* (%)	0	13 (8.6)	11 (15.1)	2 (4.3)
		1	29 (19.1)	15 (20.6)	14 (30.4)
		2	21 (13.8)	13 (17.8)	8 (17.4)
		3	20 (13.2)	12 (16.4)	8 (17.4)
		4	8 (5.3)	6 (8.2)	2 (4.3)
		5	1 (0.7)	1 (1.4)	12 (26.1)
		6	27 (17.8)	15 (20.6)	2 (4.3)
Occlusion site	*n* (%)	Cervical CI	3 (2)	2 (2.1)	1 (1.8)
		Terminal CI	7 (4.6)	0 (0)	7 (12.3)
		Carotid T	29 (19.1)	20 (21.1)	9 (15.8)
		M1	60 (39.5)	44 (46.3)	16 (28.1)
		M2	38 (25)	22 (23.2)	16 (28.1)
		A1	1 (0.7)	0 (0)	1 (1.8)
		P1	2 (1.3)	2 (2.1)	0 (0)
		P2	2 (1.3)	1 (1)	1 (1.8)
		Mixed	7 (4.6)	3 (3.2)	4 (7)
		M3	3 (2)	1 (1)	2 (3.5)
IVT - *n* (%)			89 (58.6)	48 (50.5)	41 (71.9)
EVT - *n* (%)			149 (98)	94 (98.9)	55 (96.5)
βTICI	*n* (%)	0	19 (12.5)	8 (8.5)	11 (19.3)
		1	1 (0.7)	1 (1.1)	0 (0)
		2a	9 (5.9)	4 (4.3)	5 (8.8)
		2b	38 (25)	25 (26.6)	13 (22.8)
		2c	21 (13.8)	13 (13.8)	8 (14)
		3	61 (40.1)	43 (45.7)	18 (31.6)

### Correlations

Significant correlations were observed between LBM and stroke volumes. This correlation between LBM and stroke volumes was sex-specific in the female sex. There was the expected negative correlation between LBM and age. Admission NIHSS was significantly correlated with lean body mass, only in the entire group. These correlations were not observed with BMI or 3 months modified Rankin Score (mRS).

Correlation analysis are summarized in Table 2.

**Table 2:** Spearman’s correlations between LBM or BMI and age, core, penumbra, mismatch ratio, NIHSS at admission and mRS at 3 months.

		Entire group		Women		Men	
		Rho	*p*-value	Rho	*p*-value	Rho	*p*-value
LBM with	Age	−0.175	**0.040**	−0.103	**0.336**	−0.276	0.053
	Core	0.320	**< 0.001**	0.281	**0.008**	0.080	0.579
	Penumbra	0.294	**< 0.001**	0.286	**0.007**	0.184	0.200
	Mismatch Ratio	−0.275	**0.002**	−0.240	**0.044**	−0.064	0.661
	NIHSS at admission	0.184	**0.031**	0.194	0.070	4.814 × 10^−4^	0.997
	mRS at 3 months	0.080	0.409	0.162	0.179	−0.150	0.355
BMI with	Age	−0.075	0.383	2.556 × 10^−4^	0.998	−0.238	0.096
	Core	0.088	0.302	0.171	0.109	−0.155	0.282
	Penumbra	0.131	0.125	0.162	0.130	0.020	0.892
	Mismatch Ratio	−0.048	0.591	−0.150	0.188	0.162	0,265
	NIHSS at admission	−0,005	0,953	0,044	0,686	−0,145	0,314
	mRS at 3 months	−0.027	0.783	0.005	0.965	−0.076	0.641

Statistically significant values (*p* < 0.05) are highlighted in bold.

### Lean body mass median-split

The LBM median split showed that higher LBM is associated to higher stroke volume in women. High LBM in women erases sex-differences in stroke characteristics with similar stroke volumes than men.

Table 3 details the LBM median-split analysis according to sex.

**Table 3:** Median split according to LBM’s median and comparisons of clinical characteristics.

	Entire group			Women			Men		
LBM	m ± sd		*p*	m ± sd		*p*	m ± sd		*p*
	LowLBM	HighLBM		LowLBM	HighLBM		LowLBM	HighLBM	
Age* (y)	72 ± 14	69 ± 14	0.175	71 ± 14	70 ± 14	0.639	81 ± 11	69 ± 14	0.108
Sex (W%)	93%	31%	**<0.001**						
BMI* (kg/m^2^)	25.6 ± 4.5	27.0 ± 2.9	**0.004**	25.7 ± 4.4	28.3 ± 2.6	**0.002**	24.2 ± 6.6	26.4 ± 2.8	0.140
Core* (ml)	20 ± 28	34 ± 42	**0.014**	20 ± 29	29 ± 39	0.171	18 ± 13	36 ± 44	0.465
Penumbra* (ml)	93 ± 48	123 ± 66	**0.007**	91 ± 49	126 ± 59	**0.017**	114 ± 34	122 ± 70	0.867
Admission NIHSS	15 ± 8	16 ± 7	0.144	14 ± 8	17 ± 6	0.232	17 ± 7	16 ± 7	0.881
mRS at 3 months			0.554			0.16			0.41

For continuous variables(*), Mann–Whitney’s U-test was applied. For discrete variables, *χ*^2^ test was applied. Statistically significant values (*p* < 0.05) are highlighted in bold.

### Linear regressions

Patients with an admission mRS score < 3, anterior LVO, and treated with mechanical thrombectomy were included in this study.

Significant correlations were observed between LBM and stroke volumes in both the entire group and the female subgroup (women’s core: *t* = 0.042, women’s penumbra: *t* = 0.005). In this model, LBM explains 6% (*R*^2^ entire group = 0.064 - *R*^2^ women = 0.096 - *R*^2^ men = 0.005) of penumbra’s variability, while age explains only 0.6% (*R^2^* = 0.006) of penumbra’s variability. A variation of 10 kg in LBM correlates with a proportional variation of 17 mL in penumbra.

Linear regressions of stroke volumes by BMI were evaluated. Slope for both penumbra (*t* = 0.259) and core (*t* = 0.827) were found to be statistically non-significant.

**Figure 2:**
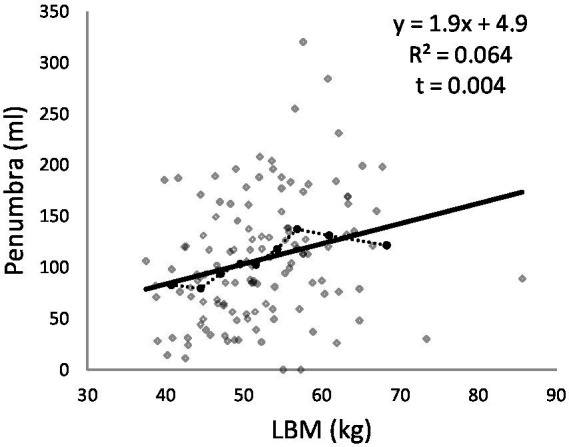
Penumbra according to LBM. In continuous line, regression curve. In dotted line, croissant class of LBM curve.

Figure 2 illustrates the regression curves for LBM and stroke volume.

## Discussion

The main findings of this study are that higher LBM is associated with higher stroke volumes in LVO and that the association between LBM and stroke volume is specific of the female sex.

Although based on a monocentric cohort, our findings are likely to be valid in LVO populations. Indeed, our cohort closely matches the clinical characteristics of other large LVO cohorts in term of age, sex, stroke risk factors, admission NIHSS and functional outcomes (1, 24–26). Similarly, perfusion parameters were within the same range in term of core and penumbra volume as in the meta-analysis performed under the HERMES collaboration that pooled patient-level data from all randomized controlled trials that compared endovascular thrombectomy with standard medical therapy (27). Furthermore, the clinical and imaging characteristics from our population also parallels the characteristics from studies that compared LVO perfusion parameters and outcomes in men and women (8, 9, 28, 29). Finally, the LBM values we report in our study correspond to the normative data for 70 years old male and females (30–32). All those facts concur to suggest that our observation apply to LVO cohorts in general.

To the best of our knowledge, no previous studies have evaluated the effect of LBM on LVO clinical and perfusion parameters at the acute phase. The finding that higher LBM is associated with higher stroke volumes seems paradoxical. Indeed increased LBM is associated with lower mortality rates in older individuals (33) as well as to larger brain grey matter volume (34) and lower rates of brain atrophy (35). In LVO, the level of brain atrophy is predictive of futile recanalization (36) and functional outcome (37). So, a variable usually associated with better brain trophicity would be expected to be beneficial instead of detrimental. The reason why LBM is associated with higher LVO stroke volume in women is probably not to be found in brain leptomeningeal collaterality as arterial collaterality scores are better in women than men in LVO (8) but could relate to tissular collaterality issues. Indeed, brain tissular collaterality is reflected by LVO stroke core and penumbra volumes and while women tend to have lower LVO stroke volumes, women with higher LBM in our study showed similar stroke volumes than men. Thus, higher LBM in women seems to erase the sex-effect in LVO stroke volumes. This observation is not due to the age difference between men and women in our study, as regression models showed that LBM explained a ten times higher proportion of penumbra volume variability than age. A possible explanation as to why higher LBM lessens the positive female sex-effect on LVO stroke volumes could relate to blood pressure levels. Increased LBM is positively correlated with elevated mean blood pressure (38, 39). Furthermore, heightened blood pressure levels exhibit an adverse association with functional outcomes following LVO and an augmented burden of white matter lesions (WML). Cerebral blood flow is more impaired with higher burden of WML (40) and WML burden is significantly higher in women compared to men (41). This cascade of events may serve as a circumstantial elucidation for the observed correlation wherein elevated LBM in females correlates with heightened perfusion parameters in both male and female subjects.

Different cerebral self-regulation mechanisms in relation to LBM could also contribute to the sex difference we report. Previous research has reported differences in the adaptability of the cardiovascular system to exercise, particularly in women, that were strongly correlated with LBM (42). Decreased LBM is associated with decreased cardiac output (CO) and increased peripheral vascular resistance, while increased LBM has shown the opposite effects on CO and peripheral vascular resistance (42). This raises the question of whether a similar phenomenon occurs at the intracranial level, influencing the myogenic tone of the arteries. During acute ischaemia, the fall in CBF induces hypoxia which favors vasodilatation, while the fall in pressure causes myogenic vasodilatation (43). In these patients, whose tone is already reduced, additional myogenic vasodilatation could be limited, effectively increasing the penumbra volume. To assess this hypothesis, it would be of interest to evaluate CBF using transcranial Doppler during the acute phase of LVO stroke as well as post-stroke, considering potential gender differences in CBF, and their association with varying levels of LBM.

Finally, BMI, owing to its role in defining obesity, is widely used to describe the weight status of patients. However, its utility is limited when it comes to assessing stroke volume or predicting the functional outcome of patients. This limitation may be attributed, at least in part, to the lack of representativeness of body composition in its calculation (44).The use of LBM could potentially serve as a more suitable tool for assessing body composition in stroke patients. One could object that in our study, we used James’ equation to compute LBM and not Dual X-ray Absorptiometry (DXA) that is the gold-standard to assess body composition. However, DXA is not suited for acute stroke settings and LBM estimation equations, such as James’, displays 0.948 Pearson rank correlation with DXA measures (45) suggesting that LBM equation are effective surrogates to estimate LBM.

In summary, we showed that LBM has a sex specific effect on LVO stroke volumes in women. This observation warrants further studies to understand the pathophysiology behind this association and help understand why women fare worse after an LVO stroke than men in order to provide tailored care.

## Data Availability

Data can be shared upon reasonable request. Requests to access the data should be directed to Gilles Naeije (gilles.naeije@hubruxelles.be).
